# The impact of the new Dutch law on Court-Orders or Care Authorizations: Less or Shorter involuntary Admissions due to Earlier Intervention?

**DOI:** 10.1192/j.eurpsy.2025.486

**Published:** 2025-08-26

**Authors:** M. Lungu, S. Gemsa, E. Noorthoorn

**Affiliations:** 1 GGNet, Warnsveld, Netherlands

## Abstract

**Introduction:**

As of January 1, 2020, the new Dutch Mental Health Compulsory Care Act (WVGGZ) came into effect, replacing the Special Admissions to Psychiatric Hospitals Act (Wet BOPZ).

**Objectives:**

One of the expectations of the WVGGZ was that earlier (medication-based) involuntary intervention in outpatient settings would lead to fewer or shorter hospital admissions. The most important change was the outpatient use of involuntary medication, which was not allowed before the law.

**Methods:**

Data from the Argus rating scale (Noorthoorn et al, 2016) and its successor, the compulsory care registration were combined with background data on admissions and wards. For this study, patients with a court order (RM) in 2016 and 2017 and a cumpulsory care authorization (ZM) in 2022 and 2023 were selected.

**Results:**

In 2016 and 2017, 549 patients received an RM, and 958 RM’s were issued. In 2022 and 2023, there were 405 patients, and 546 ZM’s were implemented. The total number of admission days for these patients before the law was 78,183 compared to 76,099 after the law. The average length of stay increased from 81 days per patient to 140 days per patient (t=-8.93, p<0.001). Seclusion decreased from 45,893 hours to 2,985 hours (t=4.93, p<0.001). Intramuscular (IM) medication was administered 137 times before the law and 369 times in clinical settings after the law (2022-2023) (chi-square= 325.10, p<0.001). IM medication was administered 777 times in outpatient settings.

**Conclusions:**

Contrary to expectations, the legislative change did not result in shorter admissions for patients with a ZM. The data indicate significant differences in involuntary treatment for patients before and after the legal change. The presentation will explore possible factors influencing these figures.

**Disclosure of Interest:**

None Declared

